# Does social support improve self-management among rural hypertensive patients? An empirical analysis based on generalized propensity score matching

**DOI:** 10.3389/fpubh.2024.1445946

**Published:** 2025-01-07

**Authors:** Jiantao Li, Jingru Zhang, Yuxiao Wang, Hanwen Zhang, Yangyang Ma

**Affiliations:** ^1^Department of Health Economics, School of Management, Shanxi Medical University, Taiyuan, China; ^2^Academy of Medical Sciences, Shanxi Medical University, Taiyuan, China

**Keywords:** hypertension, social support, self-management, generalized propensity score matching, causal effect

## Abstract

**Background:**

This study aimed to examine the causal effect between perceived social support and self-management in rural patients with hypertension and to provide a basis for improving self-management.

**Methods:**

A cross-sectional study of 1,091 rural hypertensive patients in Shanxi Province was conducted from March through June 2022 to analyze the factors influencing social support as well as the causal effects of social support and self-management using generalized propensity score matching.

**Results:**

Rural hypertensive patients had a low level of social support (social support score = 0.632 ± 0.178). Social support had a significant and inverted U-shaped relationship with self-management; with increasing social support levels, the levels of self-management first rose and then declined, with an inflexion point of 0.774. Social support had significant negative correlations with sex, age, number of child, living status (i.e., living alone or living with others), disease duration, family economic status, and decision-making power, and positive correlations with having a spouse and having medical insurance.

**Conclusion:**

Greater emphasis should be placed on the older adult, individuals living alone, those without spouses, only children, economically disadvantaged populations, and uninsured individuals to enhance the social support they received and ultimately improve their self-management of hypertension. Furthermore, establishing social support systems that are congruent with rural relational networks is crucial for promoting effective hypertension self-management.

## Introduction

Hypertension (defined as systolic blood pressure or diastolic blood pressure at 140/90 mmHg or higher) is one of the most common chronic diseases globally and can increase the risk of heart, brain, kidney and other diseases (1, 2). Global Report on Hypertension 2023 published by the World Health Organization showed that the estimated global prevalence of hypertension among people aged 30–79 years is 33% and more than 1 billion people (82%) lived in lower-middle-income areas (3). Evidence from the China Patient-centered Evaluative Assessment of Cardiac Events indicates that, of the more than 1.7 million people in the 35–75 age range who participated in the survey, approximately 44.7% had hypertension, with 63.1% of hypertensive patients coming from rural areas (4). Hence, hypertension is a major public health concern in China.

The use of long-term anti-hypertensive medications under medical supervision is effective in controlling elevated blood pressure and reducing the risk of cardiovascular disease and all-cause mortality (5–7). However, in rural areas with limited health resources, individuals with hypertension have limited access to sustained and effective anti-hypertensive treatments. The Revised Chinese Guidelines for the Management of Hypertension 2024 show that in recent years, the rising trend of hypertension prevalence in China’s rural areas has been more pronounced than in urban areas. The awareness, treatment and control rates of hypertension in China are 51.6, 45.8, and 16.8% respectively, which are still relatively low (8). Thus, prevention and treatment of hypertension in rural areas should receive more attention.

Self-management is the ability of patients to initiate healthy behaviors, monitor and manage disease progression, and promote their health (9). Given the large population of rural hypertensive patients, self-management is a suitable technique for chronic disease prevention and control based on China’s national health conditions (10). Self-management of hypertension in rural areas is carried out by primary healthcare institutions (e.g., township health centers) led by village doctors and in the form of standardized self-management group activities (11). The implementation of self-management has effectively reduced blood pressure and improved patients’ quality of life (12, 13).

Social support (SS) refers to the material or mental assistance that individuals receive through social interactions (14). SS is closely intertwined with both physical and mental well-being and serves as a crucial moderating factor in the relationship between stress and health, helping people counteract the negative effects of stress (15, 16). Studies on disease self-management have also identified SS as having an important positive influence on self-management (17–19). A finding from the Jackson Heart Study (African American Community Cohort) indicated that a high level of functional social support was associated with lower risk of incident hypertension (20). However, an in-depth exploration of the relationship between SS and self-management has revealed inconsistent associations. For instance, a study analyzing the role of SS in self-management activities in diabetic patients found that emotional, instrumental, and functional support were significantly and positively associated with self-management activities such as exercise, diet, and medication use, and emotional SS was negatively associated with foot care activities (21). Moreover, belonging to a social network has a potentially negative impact on self-management (22). Faced with these different outcomes, we debated whether SS promotes self-management.

Prior research has primarily employed structural equation modeling (23), logistic regression analysis (24), and mediation effect analysis (25) to examine the factors influencing self-management. Nevertheless, there may be additional confounding variables that impact the relationship between social support and self-management. Furthermore, it is possible that a threshold exists for the impact of social support on self-management; specifically, beyond a certain level, excessive social support may act as a hindrance for patients. As such, we investigated the role of SS in the self-management of patients with hypertension in rural China. We developed a generalized propensity score matching (GPSM) model to assess the dose–response and marginal effects of SS and self-management. Our findings provide novel insights for the provision of SS interventions to improve self-management in patients with hypertension.

## Methods

### Research settings

We conducted this study between March and June of 2022 at the Department of Health Economics, which is part of the School of Management at Shanxi Medical University. We employed a multi-stage stratified cluster sampling method based on the geographic distribution of 11 municipal areas in Shanxi Province, categorizing them into north, central, and south regions. From each region, one municipal unit was randomly selected, followed by the random selection of three counties or districts within that unit. Subsequently, one township was randomly chosen from each county or district, and then three administrative villages were randomly selected from each township. Ultimately, the final survey units comprised nine villages. We selected the entire cluster of hypertensive patients registered at the village clinic for the questionnaire survey. The inclusion criteria were as follows: (1) The patient met the diagnostic criteria of the Chinese Guidelines for the Management of Hypertension (Revised 2024) (8). (2) The patient had resided permanently in the village for at least 1 year. (3) There were no communicative barriers and the patient participated voluntarily in the survey. The exclusion criteria entailed a lack of comprehension, hearing problems, and mental disorders. Under the coordination of the health administrative department of the sampling area, township health centers and village doctors organized the sampled patients to complete the questionnaire at the village health center. Given the limited literacy and other constraints facing rural patients, the researcher was trained to fill out the questionnaire using one-to-one questions and answers to ensure that all participants fully understood the study’s requirements.

### The sample capacity

The sample capacity estimation formula for single sample mean was used to calculate the sample size (26). The formula was calculated as follows:


$$N=\frac{Z_{\alpha /2}^{2}\times P\left(1-P\right)}{E^{2}}$$

$Z_{\alpha /2}^{2}$ is the quantile of the standard normal distribution corresponding to the desired confidence level. The *Z*-value is about 1.96 for the 95% confidence level. E represents an acceptable margin of error, with a maximum allowable value of 0.05 (In our study, E = 0.03). P denotes the proportion of the total population exhibiting a specific characteristic. According to the China Cardiovascular Health and Disease Report 2023, the prevalence of hypertension among adults was reported at 31.6% (27), so we set *p* = 0.316. The theoretical sample size was calculated to be about 923. In our study, a total of 1,137 questionnaires were answered; we excluded 46 erroneous questionnaires. After logical verification, we included 1,091 valid questionnaires (validity rate: 95.87%), which provides sufficient statistical power to ensure the representativeness of the results.

### The questionnaire

The questionnaire comprised three parts. The first contained a section on basic information including sex, age, if the respondent had a spouse, the number of children the respondent had, his/her living status, if the respondent had medical insurance, family economic level, disease duration, and decision-making power. The second part involved the Hypertension Self-Management Scale developed by Zhao Qiuli (28), which we used to measure the level of self-management in patients with hypertension; it includes six dimensions: (1) medication, (2) diet, (3) work and rest, (4) emotions, (5) the monitoring of one’s condition, and (6) exercise management. The scale consists of 32 entries, with a total score of 160 (Cronbach’s alpha = 0.914). The standardized self-management score = the self-management score/165 × 100. The third part involves the Social Support Scale developed by Xiao Shuiyuan (29), which includes three dimensions: (1) objective support, (2) Subjective support, and (3) utilization of social support. Objective support is mainly comprised of material assistance and direct services such as blood pressure measurements, health education, and medical information from village doctors. Subjective support primarily entails experiencing emotional support such as companionship from family members, concern for one’s neighbors, and encouragement among patients. Utilization of SS refers to the active use of various forms of SS by hypertensive patients, including patient-initiated help-seeking, active confiding, and active participation in activities. The scale consists of 14 items, with a total score of 66 (Cronbach’s alpha = 0.940). The SS scalar score = the self-assessment score/66 × 100. A score below 0.33 indicates a low level of social support, 0.33–0.67 indicates a moderate level, and 0.68–1.00 indicates a high level.

### Generalized propensity score matching

It is difficult to scientifically assess and quantify the impact of SS on the level of self-management because whether a patient is affected by SS is a non-random event and is impacted by various confounding factors such as sex, age, and economic level (30, 31). To eliminate this confounding bias, Rosenbaum and Rubin proposed a propensity score matching (PSM) approach based on a counterfactual framework that effectively reduced covariate-induced bias by calculating conditional treatment probabilities for covariates and then balancing covariates in the intervention and control groups (32). However, traditional PSM models can only test the effects of dichotomous treatment variables, which significantly limits their scope. To cope with the causal inference of continuous treatment variables, Hirano and Imbens improved the PSM model and proposed GPSM (33). As such, we used GPSM to explore the effects of SS on self-management.

GPSM estimates treatment effects in three steps. First, we estimated the conditional probability density of the treatment variable (i.e., SS) and analyzed the influencing factors. Because the treatment variable failed the normality test (after adjustment via log transformation, Box-Cox transformation, etc.), we employed the fractional logit model proposed by Guardabascio and Ventura (34). To satisfy the conditions for the usability of the fractional logit model, we centered the treatment variable such that the value of this variable would fall within the interval [0, 1]. Second, we estimated the conditional distribution of the outcome variable (i.e., self-management). Last, we estimated the dose–response and treatment effect functions for the treatment variable (i.e., SS) on the outcome variable (i.e., self-management).

### Definitions of the variables

We expressed the outcome variable (i.e., self-management) as a standardized score on the Hypertension Self-Management Scale. We expressed the treatment variable (i.e., SS) as a standardized score on the Social Support Scale. We normalized both the outcome and treatment variables to eliminate the effects of scale and outliers. The covariates included sex, age, spouse, number of child, living conditions, type of medical payment, family economic situation, disease duration, and decision-making power. Table 1 defines the variables.

**Table 1:** Definitions and descriptions of the variables.

Category	Name	Description
Outcome	Self-management	This variable measures the level of self-management among rural hypertensive patients, adjusted by data normalization to exclude the effects of dimensions and outliers.
Treatment	Social support	This variable measures the level of social support among rural hypertensive patients, adjusted for data normalization to exclude the effects of the scale and outliers.
Covariate	Sex	1 = male; 0 = female
	Age	1 = age ≥ 60; 0 = age<60
	Spouse	1 = with; 0 = without
	Number of child	Number of children of the respondents. 1 = only child; 0 = not the only child
	Living status	1 = live alone; 0 = living with others
	Medical insurance	1 = with; 0 = without
	Family economic status	1 = poor family; 0 = non-poor family
	Disease duration	1 = disease duration>10 years; 0 = disease duration≤10 years
	Decision-making power	1 = with primary decision-making power; 0 = without

### Statistical analysis

We used Stata16 for statistical analyses. In this study, we employed descriptive statistics to analyze the demographic traits of the respondents as well as the self-management and SS scores of rural hypertensive patients. We employed a GPSM model to estimate the dose–response and treatment effects of SS on self-management.

## Results

### Description of the variables and the characteristics of the population

As presented in Table 2, 1,091 rural patients with hypertension participated in the survey. In this sample, most of the respondents were female (67.83%), old (age ≥ 60) (77.91%), had a spouse (81.12%), were not the only children (98.35%), lived with others (86.89%), had medical insurance (73.05%), came from a poor family (67.92%), has a disease duration of 10 years or longer (95.05%), and did not have decision-making power (59.21%). The normalized adjusted standardized self-management score for rural hypertensive patients was 0.541 ± 0.150 and the SS score was 0.632 ± 0.178 (Table 3).

**Table 2:** The characteristics of rural hypertensive patients.

Characteristic	*N* = 1,091	%
Sex		
Male	351	32.17
Female	740	67.83
Age		
<60	241	22.09
≥60	850	77.91
Spouse		
With	885	81.12
Without	206	18.88
Number of child		
Only child	18	1.65
Not the only child	1,073	98.35
Living status		
Live alone	143	13.11
Living with others	948	86.89
Medical insurance		
With	797	73.05
Without	350	32.08
Family economic status		
Poor family	741	67.92
Non-poor family	350	32.08
Disease duration		
Disease duration>10 years	1,037	95.05
Disease duration≤10 years	54	4.95
Decision-making power		
With	445	40.79
Without	646	59.21

**Table 3:** Descriptive statistics: the outcome and treatment variables.

Variable	*n*	M ± SD	Min	Max
Self-management	1,091	0.541 ± 0.150	0.064	1.000
Social support	1,091	0.632 ± 0.178	0.101	1.000

### Social support: fractional logit regression

Based on the fractional logit model, we obtained coefficient estimates for the conditional distribution function of the level of SS. As seen in Table 4, SS significantly correlated with all nine covariates. Among them, it was negatively correlated with seven covariates in descending order of strength: number of child (coef. = −0.455, *p* < 0.05), living status (coef. = −0.335, *p* < 0.001), disease duration (coef. = −0.231, *p* < 0.001), sex (coef. = −0.164, *p* < 0.001), family economic status (coef. = −0.108, *p* < 0.05), age (coef. = −0.106, *p* < 0.05), and decision-making power (coef. = −0.082, *p* < 0.05). SS was positively associated with having a spouse (coef. = 0.236, *p* < 0.001) and having medical insurance (coef. = 0.201, *p* < 0.001).

**Table 4:** Results of the estimation of factors influencing social support based on fractional logic regression.

Dependent variable: Social support	Coef.	*Z*	*p*	95% CI	
				Low	Upper
Sex	−0.164	−4.03	0.000^***^	−0.243	−0.084
Age	−0.106	−2.17	0.030^**^	−0.203	−0.010
Spouse	0.236	3.51	0.000^***^	0.104	0.367
Number of child	−0.455	−2.04	0.041^**^	−0.892	−0.018
Living status	−0.335	−4.01	0.000^***^	−0.499	−0.171
Medical insurance	0.201	4.70	0.000^***^	0.117	0.285
Family economic status	−0.108	−2.69	0.007^**^	−0.186	−0.029
Disease duration	−0.231	−3.33	0.000^***^	−0.367	−0.095
Decision-making power	−0.082	−2.16	0.031^**^	−0.157	−0.007
const.	0.338	2.97	0.003^**^	0.115	0.561

***, **, * represent significance at the 1, 5, and 10% levels. CI, confidence interval.

### Verifying the balance condition

Based on the estimated conditional distribution of SS, we computed and matched propensity score values. Successful matches require a balanced condition. The test was designed to ensure that there would be no confounding characteristics other than SS that differed significantly between the treatment and control groups. In addition, appropriately matched grouping and segmentation of the samples are required to achieve a balanced condition. Because SS is biased toward the 0-value end of the [0, 1] interval, we attempted to subdivide the sample into five groups by selecting treatment intensities of 0.255, 0.447, 0.553, 0.638, and 0.745 as critical values. We further divided the groups into five segments based on their average generalized propensity score (GPS). Table 5 presents the results of the verification of the balance condition. When unadjusted, all covariates were significantly different. After adjusting for GPSM, most covariates did not pass the test for differences in t-values, which indicated that there was no longer a significant difference in sample characteristics between the control and treatment groups. Thus, the balance condition was satisfied.

**Table 5:** Verification of the balance condition: t-statistics for equality of means.

Covariate	Unadjusted	Adjusted for the GPS					
		[0, 0.255]	(0.255, 0.447]	(0.447, 0.553]	(0.553, 0.638]	(0.638, 0.745]	(0.745, 1]
Sex	0.059^***^ (6.234)	−0.024 (−0.23)	−0.050 (−1.43)	−0.037 (−1.40)	0.006 (0.18)	−0.001 (−0.03)	0.077 (0.93)
Age	0.054^***^ (5.046)	−0.077 (−0.74)	−0.071^*^ (−1.86)	−0.035 (−1.25)	−0.040 (−1.47)	0.030 (1.03)	0.122^**^ (2.02)
Spouse	−0.131^***^ (−12.083)	0.007 (0.13)	0.002 (0.08)	0.081^**^ (3.12)	−0.014 (−0.53)	−0.112^**^ (−3.21)	−0.142^*^ (−1.85)
Only child	0.246^***^ (7.077)	−0.024^**^ (−3.17)	−0.006 (−0.64)	0.000 (−0.02)	0.020^*^ (1.87)	0.018 (1.38)	0.017 (0.59)
Living condition	0.164^***^ (13.163)	0.024 (1.26)	−0.018 (−1.23)	−0.078^***^ (−3.39)	0.079^**^ (3.22)	0.079^**^ (2.48)	0.134^*^ (1.91)
Medical insurance	−0.067^***^ (−6.705)	0.159^*^ (1.65)	−0.001 (−0.03)	0.001 (0.02)	−0.023 (−0.77)	−0.005 (−0.13)	0.185^**^ (2.27)
Family economic situation	0.053^***^ (5.574)	−0.256^**^ (−2.22)	0.102^**^ (2.50)	−0.060^*^ (−1.94)	−0.041 (−1.42)	−0.025 (−0.83)	−0.011 (−0.16)
Illness	0.062^***^ (2.986)	−0.169 (−1.48)	−0.012 (−0.32)	−0.035 (−1.08)	0.045 (1.37)	0.078^*^ (1.90)	0.047 (0.55)
Decision-making power	0.059^***^ (6.509)	−0.047 (−0.85)	−0.048^**^ (−2.22)	0.009 (0.57)	0.006 (0.43)	−0.015 (−0.99)	0.048^*^ (1.75)

***^,^ **^,^ * represent significance at the 1, 5, and 10% levels.

### Estimating the conditional distribution of the outcome variable (i.e., self-management)

Based on the estimation of the conditional distribution of SS and the measure of the GPS value, we estimated the conditional distribution of the outcome variable (i.e., self-management). Table 6 displays the results. The significance of the regression coefficients indicates that the model is a good estimator of conditional self-management distribution. SS (Coefs. = 4.080, *p* < 0.001) and GPS (coef. = 1.134, *p* < 0.05) were significantly and positively associated with self-management.

**Table 6:** Results of estimating the conditional distribution of self-management.

Dependent variable: Self-management	Coef.	*Z*	*p*	95% CI	
				Low	Upper
SS	4.080	3.19	0.000^***^	1.567	6.594
SS^2^	2.364	−3.04	0.002^**^	−3.893	0.836
GPS	1.134	2.33	0.020^**^	0.181	2.087
GPS^2^	0.244	−2.27	0.023^**^	−0.455	−0.033
SS × GPS	1.581	−2.29	0.022^**^	−2.937	0.225
_cons	−0.945	−1.81	0.071^*^	−1.970	0.081

***, **, * represent significance at the 1, 5, and 10% levels.

CI, confidence interval; GPS, generalized propensity score; SS, social support.

### Dose–response and treatment effect functions

**Figure 1:**
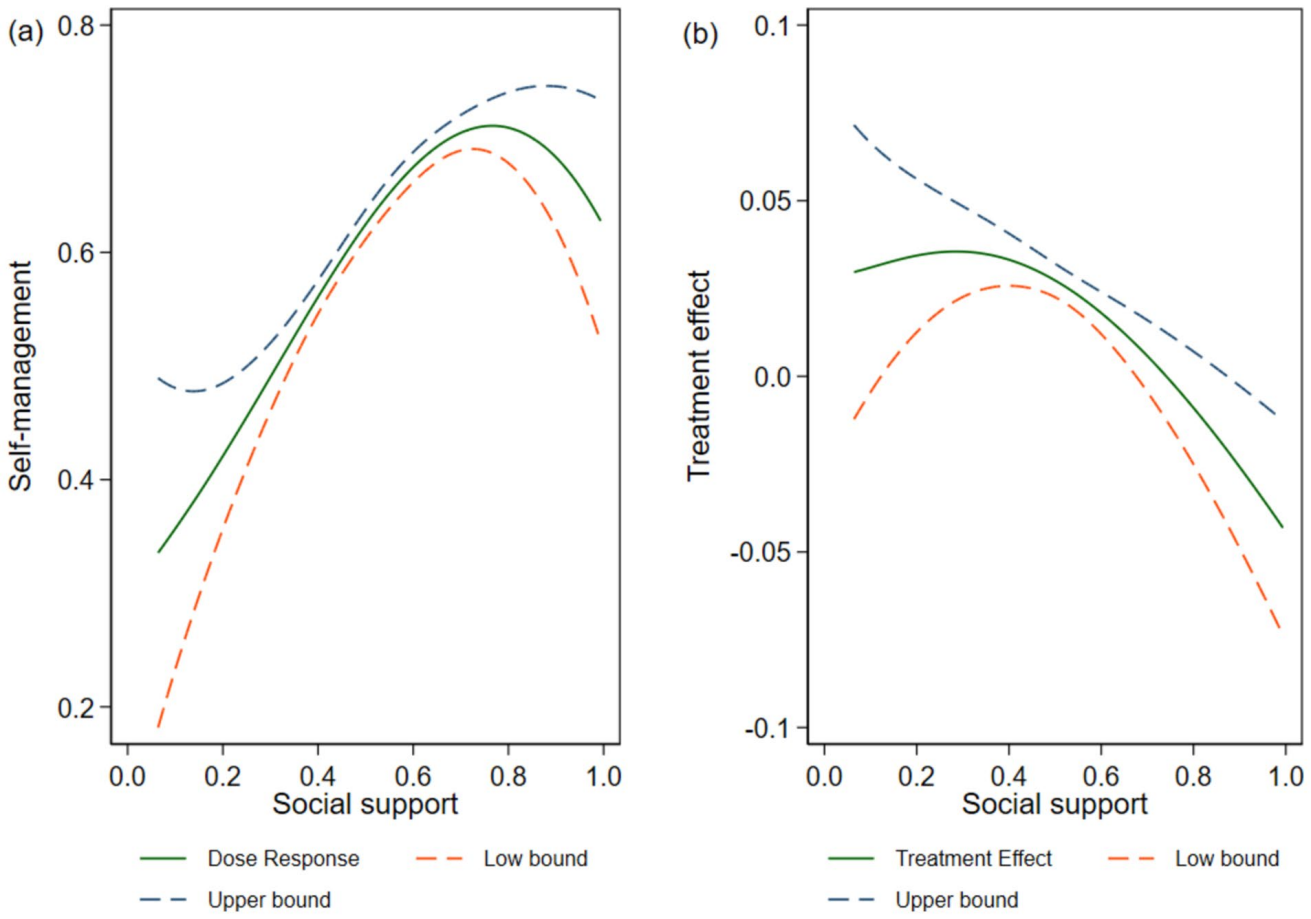
Social support and self-management; **(A)** average dose–response function and **(B)** treatment effect function.

Figure 1 illustrates the net effect of SS on self-management. Figure 1A shows the average dose–response function of SS in self-management, and Figure 1B illustrates the treatment-effect function of SS. We assessed the confidence intervals (CIs) for both via bootstrapping and they are indicated by the upper and lower dashed lines. Figure 1A suggests that there is a significant and inverted U-shaped relationship between SS and self-management: With increasing levels of SS, self-management levels first rise and then fall. Figure 1B portrays this phenomenon. SS had a positive effect on self-management when it was less than 0.774. However, when it was greater than 0.774, it had an inhibitory effect on self-management.

## Discussion

We examined the relationship between SS and self-management based on questionnaire data collected from rural patients with hypertension. We analyzed the conditional distribution of SS in conjunction with GPSM, based on which we calculated the GPS. We divided the participants into six groups based on their GPS, which eliminated the confounding effects of sex, age, having a spouse, family economic status, and disease duration. Finally, we sketched the dose–response curve of SS and self-management, the marginal effect curve of SS, and we quantified the relationship between SS and self-management. We innovatively applied the GPSM model to the field of social medicine to provide a new way of quantitatively analyzing the effects of social interventions. These findings provide new insights into the provision of SS interventions to improve self-management in patients with hypertension.

The results of the factors that influence SS, based on fractional logic regression estimation, showed that SS was negatively associated with number of child, living status, disease duration, sex, family economic status, age, and decision-making power, and positively associated with having a spouse and health insurance. This implies that the level of SS in the population of only children is lower than that in the population of people with siblings, and that the care of family members (especially children) is the main source of SS for chronic patients; this is consistent with the results of related studies (35). People who live alone have lower levels of SS due to the increased health risks associated with loneliness (36, 37). In our study, we found that people with a disease duration of 10 or more years had worse perceptions of SS and less access to it. This may be because pain caused by long-term illness can easily lead to anxiety, depression, and other negative emotions caused by chronic ailments (38). Men had lower levels of SS than women, which is consistent with the findings of related studies (39, 40). This is primarily because women’s perceived SS comes from seeking and receiving emotional support, whereas men are more concerned with tangible support (41). The availability of SS is closely tied to the economic status of one’s family. We found that poor families receive less SS than non-poor families, which may be due to economic conditions that limit access to SS such as medication, health training, and medical check-ups (42). In terms of age, older people have low levels of SS, which is consistent with the literature (43–45). In terms of household decision-making power, those with primary decision-making power in the home have low levels of SS, which is a different outcome from the results of previous studies (46, 47). In addition, SS was much higher in the spousal population than in the non-spousal population, which aligns with the findings of pertinent studies (48). The higher level of SS among individuals with medical insurance may be because medical insurance reimburses hypertensive patients for the cost of treatment (49); this increases their willingness to seek medical attention (50) and provides them with an opportunity to obtain more SS. Hence, providers of SS should focus on key populations such as those without spouses, those who have only children, those who live alone, those who are poor, and those without health insurance.

We found that SS had a significant and inverted U-shaped relationship with self-management. When it was less than 0.774, SS had a positive impact on self-management; when it exceeded this threshold, it had a negative impact. This suggests that the positive effects of SS on health are limited and excessive SS can harm health and health management. This phenomenon may be attributed to the fact that excessive objective social support can enhance patients’ sense of dependence, as evidenced by a diminished sense of responsibility for self-management in dietary practices, medication adherence, emotional regulation, and physical activity. Previous studies have also found that among people with inflammatory bowel disease, widespread talk about symptoms and treatments causes discomfort and anxiety and that excessive concern from family or friends about the patient’s health increases uncertainty about the patient’s future health status, which causes him/her to cope negatively with the disease (51). In addition, Kaushansky et al. pointed out that in the self-management of chronic illness, people often refuse to disclose their condition to society due to the stigma of being sick, not wanting to be pitied, or not wishing to be seen as a person with special needs (52). Thus, in the face of excessive attention, patients must remain positive to mitigate the negative effects of SS. An accurate understanding of the disease is important for self-management of a chronic illness. Hence, providers of SS should help patients to have correct and objective knowledge of chronic ailments in order to reduce stigma.

Ultimately, establishing a social support system that aligns with the rural relational network is essential for promoting self-management of hypertension. From the perspective of social support providers, it is crucial to delineate the pivotal role of intergenerational support from children within the social support network of rural patients. In regions where family-based older adult care predominates, intergenerational assistance provided by adult children constitutes nearly all available social support for older adult individuals. Furthermore, empathetic peer support among patients has a distinctive enhancing effect (53); communication between patients can facilitate better adaptation to their roles in managing chronic conditions (54). Using a skills training model with healthcare staff teaching, group leader leading, and group members discussing and practicing, chronic disease self-management measures are promoted. Finally, individuals are primarily responsible for their own health. Hypertensive patients need to actively manage diet, exercise, and emotional health.

Finally, the application of the GPSM to evaluate the effectiveness of the social interventions examined was reliable. The findings of the equilibrium condition test indicated that the GPSM balanced covariate differences between the groups. The results of the self-management conditional response function, estimated based on the GPSM model, revealed that the independent variables passed the 1, 5, and 10% significance tests, and the model fit was good.

However, this study has some limitations. First, we administered it only to rural patients with hypertension, which may limit the generalizability of our findings to the broader population. Second, the measures of SS and self-management were based on patients’ self-perceptions; thus, there may have been a degree of bias. Third, the sample size was small and needs to be validated with a larger sample to improve the reliability and validity of the findings. Finally, owing to the research conditions, we conducted a cross-sectional survey; future studies should include longitudinal studies to more comprehensively understand the influence of SS on self-management.

## Conclusion

In conclusion, our findings indicate that the relationship between social support and self-management levels in hypertensive patients exhibits an inverse U-shaped pattern. Moreover, the indiscriminate provision of social support does not result in the sustained enhancement of self-management capabilities. Additionally, the survey results indicate that the level of social support among hypertensive patients in rural Shanxi is moderate. In the future, it is essential to prioritize vulnerable populations, including the older adult, individuals living alone or without a spouse, only children, economically disadvantaged individuals, and those lacking medical insurance. Lastly, it is of the utmost importance to establish social support systems that are congruent with rural relational networks in order to foster effective self-management of hypertension.

## Data Availability

The raw data supporting the conclusions of this article will be made available by the authors, without undue reservation.
